# Health spending and vaccination coverage in low-income countries

**DOI:** 10.1136/bmjgh-2020-004823

**Published:** 2021-05-05

**Authors:** Francisco Castillo-Zunino, Pinar Keskinocak, Dima Nazzal, Matthew C Freeman

**Affiliations:** 1 H. Milton Stewart School of Industrial and Systems Engineering, Georgia Institute of Technology, Atlanta, Georgia, USA; 2 Gangarosa Department of Environmental Health, Emory University, Atlanta, Georgia, USA

**Keywords:** public health, vaccines, health economics

## Abstract

**Introduction:**

Routine immunisation is a cost-effective way to save lives and protect people from disease. Some low-income countries (LIC) achieved remarkable success in childhood immunisation. Yet, previous studies comparing the relationship between economic growth and health spending with vaccination coverage have been limited. We investigated these relationships among LIC to understand what financial changes lead to childhood immunisation changes.

**Methods:**

We identified which financial indicators were significant predictors of vaccination coverage in LIC by fitting regression models for several vaccines, controlling for population density, land area and female years of education. We then identified LIC with high vaccination coverage (LIC+) and compared their economic and health spending trends with other LIC (LIC−) and lower-middle income countries. We used cross-country multi-year regressions with mixed-effects to test financial indicators’ rate of change. We conducted statistical tests to verify if financial trends of LIC+ were significantly different from LIC−.

**Results:**

During 2014–2018, gross domestic product per capita (p=0.67–0.95, range given by tests with different vaccines), total/private health spending per capita (p=0.57–0.97, p=0.32–0.57) and aggregated development assistance for health (DAH) per capita (p=0.38–0.86) were not significant predictors of vaccination coverage in LIC. Government health spending per capita (p=0.022–0.073) and total/government spending per birth on routine immunisation vaccines (p=0.0007–0.029, p=0.016–0.052) were significant positive predictors of vaccination coverage. From 2000 to 2016, LIC+ increased government health spending per capita by US$0.30 per year, while LIC− decreased by US$0.16 (significant difference, p<0.0001). From 2006 to 2017, LIC+ increased government spending per birth on routine immunisation vaccines by US$0.22 per year, while LIC− increased by US$0.10 (p<0.0093).

**Conclusion:**

Vaccination coverage success of some LIC was not explained by economic development, total health spending nor aggregated DAH. Vaccination coverage success of LIC+ was associated with increasing government health spending particularly in routine immunisation vaccines.

**Key questions**

**What is already known?**

- Prior studies that addressed the relationship between health spending and immunisation were limited to comparing countries at specific years (cross-sectional analysis) or considering a single country over multiple years, showing nuanced results as higher health spending does not always result in improved health outcomes.
- A recent cross-country study by Arsenault and colleagues found a positive association between higher national vaccination coverage and low out-of-pocket spending combined with high government spending.
- Evidence in Nepal and Rwanda show that government commitment was crucial to improve vaccination coverage.

**What are the new findings?**

- To our knowledge, our analysis is the first cross-country multi-year study to explain the association between health spending and vaccination coverage success among low-income countries.
- Higher economic development, total health spending and aggregated development assistance for health per capita were not associated with better vaccination coverage among low-income countries.
- Low-income countries that increased government health spending over time—particularly government spending in routine immunisation vaccines—were associated with vaccination coverage improvements.

**What do the new findings imply?**

- A larger economy and increasing total health spending per capita do not guarantee improved vaccination coverage in low-income countries.
- Increasing government health spending and development assistance for health on vaccines may lead to improved vaccination coverage.
- In many low-income countries, the increase in government spending on vaccines has been very low, and hence, they still depend on external funding, specifically on routine immunisation.

## Introduction

Routine childhood immunisation has been one of the most cost-effective public health interventions to save lives and protect people from disease.^1^ Investments in childhood immunisation were estimated to yield a net return 44 times greater than costs during 2011–2020, considering the value of people living longer/healthier lives and not needing treatment for vaccine-preventable diseases.^2^

Global childhood immunisation has significantly improved in the past decades, but there is still progress to be made in increasing coverage^3^ and understanding the impact of spending. External funding supporting vaccination efforts had a positive effect in third dose of diphtheria-tetanus-pertussis (DTP3) vaccination coverage from 1995 to 2004, while the effect was not significant in nations that reached a coverage greater than 65%.^4^ Gavi, the Vaccine Alliance, received US$ 7.1 billion from governments and private organisations to support immunisation and health systems of low-income countries (LIC) and lower-middle income countries (LMIC) during 2016–2020.^5^ The goal of the Global Vaccine Action Plan 2011–2020 was to reach a 90% national coverage by the end of the period.^6^ Despite the major efforts of international organisations and governments to improve vaccination coverage worldwide, DTP3 coverage has remained relatively consistent between 2010 (84%) and 2018 (86%).^7^

Organisations, such as the Institute for Health Metrics and Evaluation (IHME), WHO and UNICEF, have analysed several health financing indicators with different disaggregation levels, for example, funding by source and health focus area.^8–10^ These efforts enabled researchers to project health spending patterns, identify and track spending trends, and do multivariate analysis combined with other health outcomes.^11 12^

The objective of this study is to understand the impact of health financing indicators over time on vaccination coverage rates of LIC. We used fixed-effects and mixed-effects regression models^13^ to statistically compare LIC, regarding income, health spending and vaccine spending, per capita or per live birth. To the best of our knowledge, this is the first cross-country study that statistically analyses the differences among LIC in terms of health financing over time and vaccination coverage success.

Among LIC, we identified a subgroup (referred to as LIC+) with high-performance vaccination coverage compared with other LIC (referred to as LIC−) and LMIC. We investigated the time-varying differences of financial factors, such as development assistance for health (DAH) and government spending on health per capita, between LIC+ and LIC−; LMIC were used as benchmark.

## Methods

### Overview

This study consists of two parts, the first uses linear fixed-effects regression models to perform a cross-section analysis among LIC; without considering LMIC. The goal is to identify financial indicators that are the most significant predictors of vaccination coverage in LIC; after controlling for other variables such as population density, land area and female years of education.

The second part uses linear mixed-effects regression models to compare the financial trends over time between different groups of countries: LIC+, LIC− and LMIC (LMIC were used as a benchmark). The goal is to evaluate if the rate of change of financial indicators were significantly different between LIC+ and LIC− during the last decades.

### Data sources and processing

A summary of data sources can be found in the online supplemental table 1. We used the WHO and UNICEF estimates of national infant immunisation coverage (WU1^14^) from years 2000–2018. We considered the DTP1 and DTP3 vaccines, first dose of measles-containing vaccine (MCV1), BCG vaccine and third dose of polio vaccine (Pol3)—these vaccines were picked because they target diseases included in the Expanded Programme on Immunisation since 1977.^15^ These estimates are based on government reports that are supplemented by survey results from the published and grey literature, in addition to feedback from local experts.^16^

10.1136/bmjgh-2020-004823.supp1 Supplementary data

From the World Bank’s world development indicators (WB1^17^) we obtained countries’ population and live birth rate, and used them to calculate per live birth values. We also used gross national income (GNI) and gross domestic product (GDP) per capita. GNI values are expressed in US$ using World Bank Atlas method and GDP values are expressed in current US$. Land area (km^2^) was used to calculate population density (population/km^2^) and both indicators were used as control variables in the fixed-effects model.

As an additional control variable in the fixed-effects model, we used the female mean years of schooling from the United Nations’ human development reports (UNDP1^18^)—the average number of years of education received by woman ages 25 and older, converted from educational attainment levels using official durations of each level.

Global health spending estimates for 195 countries and territories were obtained from publicly available data (IHME1^8^). We used the total health spending per capita data disaggregated into government, out-of-pocket, prepaid private and DAH (expressed in constant 2018 US$). DAH are the financial resources for the improvement and maintenance of health, transferred from major health development agencies to LIC and LMIC. We calculated private health spending as the sum of out-of-pocket and prepaid private health spending.

DAH estimates from 1990 to 2018 were disaggregated by health focus areas (IHME2^9^). We used DAH spent on newborn and child health and more specifically spent on vaccines (expressed in constant 2018 US$). DAH on vaccines include funding for routine immunisation, new vaccines introduction and support for delivery components such as cold chain optimisation, systems strengthening and human resources. We removed values marked as duplicates by IHME and data from 2018 since they contained few preliminary estimates.

From the immunisation financing indicators (WU2^10^), from the WHO–UNICEF joint reporting form, we used total spending and government health spending on vaccines used in routine immunisation (expressed in constant 2010 US$). The spending on routine immunisation vaccines does not include delivery services nor spending on vaccines used for supplementary activities (included in DAH on vaccines estimates). We removed data from 2018 since these are self-reported by countries and have not been audited by WHO.

### Patient and public involvement

Patients were not involved.

### Selecting and grouping countries

We studied countries that were either LIC or LMIC in 2018 according to the World Bank’s income categorisation. GNI per capita was US$1025 or less for LIC (31 countries), and between US$1026 and US$3995 for LMIC (47 countries). We removed countries with no World Bank income category nor GNI per capita reported during 2000–2018. To avoid over-representing smaller nations, we removed countries whose population had never reached 1 million people; the latter criteria only removed countries from LMIC and not from LIC. These criteria resulted in the selection of 24 LIC and 36 LMIC for the analyses.

For the mixed-effects models, we defined subgroup LIC+ among LIC by selecting countries with a mean DTP3 coverage above 90% during 2014–2018; matching the 90% DTP3 coverage goal of the Global Vaccine Action Plan.^6^ We picked DTP3 coverage as a grouping criteria because it is widely used as proxy of routine vaccine system performance, it has always been part of the Expanded Programme on Immunisation,^15^ and indicates the completion of the initial routine immunisation.^19^ This criteria leads to three country groups: LIC+, LIC− and LMIC as summarised in online supplemental table 2.

Gavi-supported countries pay a portion of their vaccine costs depending on each country’s GNI per capita. Gavi’s co-financing model splits countries into four groups, decreasing Gavi funding from left to right: initial self-financing, preparatory transition, accelerated transition and full self-financing. LIC group closely resembles countries in the ‘initial self-financing’ Gavi group of 2018,^20^ so they received similar levels of Gavi funding; except for Tajikistan in LIC+ and Yemen in LIC− that belong in Gavi group ‘preparatory transition’. Only two countries in LMIC are in the ‘initial self-financing’ group: Senegal and Zimbabwe. Online supplemental table 2 shows the Gavi group of each LIC and LMIC studied country.

### Cross-Sectional comparison of LIC

We conducted a cross-sectional analysis among LIC to determine which financial indicators are statistically significant predictors of vaccination coverage. We used fixed-effects models with vaccination coverage as dependent variable—testing vaccines DTP1, DTP3, MCV1, BCG and Pol3 as robustness checks. We tested each financial indicator as an independent variable separately to compute p values and check for significance. The independent variables tested were GNI per capita, GDP per capita, total/government/private health spending per capita, DAH per capita, DAH on newborn and child health vaccines per live birth, total/government spending on routine immunisation vaccines per live birth.

Control variables were used in all fixed-effects regression models, including the demographic and geographic indicators of population density, land area and female mean years of schooling. Prior studies found that higher population density, smaller land area and more years of female schooling are associated with higher vaccination coverage.^21 22^

We used logistic-logarithmic transformed regression models since the logistic function normalises the dependent variables (vaccination coverage is a percentage) and logarithmic transformations normalise the independent and control variables. Variables were averaged through 2014–2018 to reduce within country variability; this year range also matches the DTP3 grouping criteria in the mixed-effects models.

### Comparing groups of countries’ rate of change

We compared LIC+ with LIC− and LMIC to understand the relationship between income and health spending rate of change and vaccination coverage success. LMIC was used as a benchmark for comparison (considering their higher levels of spending). The data used in the models span the years 2000–2018 (subject to data availability per country, summarised in online supplemental table 1), aligning with the launch of the Millennium Development Goals^23^ and the creation of Gavi in year 2000. We used mixed-effects models^13^ that enable regression analysis with correlated variables, in this case by considering the random-effects of countries, and are unbiased estimators when data are missing at random^24^ (online supplemental table 1 shows the percentage of data points missing for each variable). Fixed-effects were implemented for each country group and year period, to enable the comparison of trend rate of change (slopes) between LIC+, LIC− and LMIC.

For significance testing, we used three different approaches: an asymptotic χ^2^ test, a Kenward-Roger approximation for F tests for reduction of mean structure and a parametric bootstrap method.^25^ We computed two kinds of p values with each of the three approaches, to determine any significant differences between the group trends of LIC+ versus LIC−. First, we computed the p values of the slope coefficients, seeing if rate of change between country groups were statistically different. Second, we computed the p value of all coefficients combined—incorporating the intercept plus slope of the trends. The code implementation of the mixed-effects models and significance testing can be found in the online supplemental materials.

#### Mixed-Effects models

The linear mixed-effects models used can be formulated as follows:

(1) $$var_{tj}=\beta_{0j}+t\beta_{1j}+R_{tj}$$

for each year *t* and country *j.*

(2) $$\beta_{0j}=\alpha_{00}+U_{j}$$

for each country *j* in LIC+.

(3) $$\beta_{0j}=\alpha_{00}+\alpha_{01}+U_{j}$$

for each country *j* in LIC−.

(4) $$\beta_{0j}=\alpha_{00}+\alpha_{02}+U_{j}$$

for each country *j* in LMIC.

(5) $$\beta_{1j}=\alpha_{10}$$

for each country *j* in LIC+.

(6) $$\beta_{1j}=\alpha_{10}+\alpha_{11}$$

for each country *j* in LIC−.

(7) $$\beta_{1j}=\alpha_{10}+\alpha_{12}$$

for each country *j* in LMIC.

(8) $$R_{tj}∼N(0,\sigma^{2})$$

for each year *t* and country *j.*

(9) $$U_{j}∼N(0,\tau^{2})$$

for each country *j.*

Equation (1) represents the linear regression of the tested variable dependent of time. The data value in year *t* of country *j* is represented by *var_tj_*. Coefficients *β_0j_* and *β_1j_* are the intercept and slope, respectively, of each country *j*, which changes depending on which group they belong to. Random variables *R_tj_* illustrate the random noise within samples, that have a normal distribution with mean 0 and variance *σ*^2^ as shown in equation (8).

Equations (2)–(4) represent the intercept coefficients of LIC+, LIC− and LMIC, respectively. LIC+ have a base intercept *α*_00_, then each LIC− and LMIC have their own differences from the base intercept (coefficients *α*_01_ and *α*_02_, respectively).

Equations (5)–(7) represent the slope coefficients of LIC+, LIC− and LMIC, respectively. LIC+ have a base slope *α*_10_, then each LIC− and LMIC have their own differences from the base slope (coefficients *α*_11_ and *α*_12_, respectively). Random variables *U_j_* are random-effects that consider intercept variations within each country; they have a normal distribution with mean 0 and variance *τ^2^* as seen in equation (9). We computed p values, SEs and CIs for LIC+ and LIC−.

## Results

After fitting the cross-section fixed-effects models for 2014–2018 averages, as summarised in table 1, government health spending per capita (p=0.022–0.073, range given by tests with different vaccines) and total/government spending per birth on routine immunisation vaccines (p=0.0007–0.029, p=0.016–0.052) show up as positively associated and statistically significant predictors of vaccination coverage. Other indicators such as GDP/GNI per capita (p>0.67, p>0.53), total/private health spending per capita (p>0.57, p>0.32) and DAH per capita (p>0.38) did not show up as significant. DAH per birth on newborn and child health in general was not associated with improved vaccination coverage (p>0.47), although it was almost significant when considering only DAH on vaccines (p=0.076–0.33).

**Table 1:** Summary of cross-section fixed-effects regression models

Financial indicator	P values					Coefficients (SE)				
	DTP1	DTP3	MCV1	BCG	Pol3	DTP1	DTP3	MCV1	BCG	Pol3
GDP per capita	0.94	0.95	0.74	0.67	0.86	−0.05 (0.61)	0.03 (0.58)	0.20 (0.59)	0.31 (0.70)	−0.10 (0.56)
GNI per capita	0.94	0.85	0.60	0.53	0.99	0.04 (0.58)	0.10 (0.55)	0.30 (0.55)	0.42 (0.65)	0.00 (0.53)
Total health spending per capita	0.57	0.85	0.80	0.82	0.97	0.34 (0.59)	0.11 (0.57)	0.15 (0.58)	0.16 (0.68)	0.02 (0.54)
Government health spending per capita	**0.034**	**0.022**	**0.042**	0.073	**0.025**	1.10 (0.48)	1.11 (0.44)	1.02 (0.47)	1.08 (0.57)	1.05 (0.43)
Private health spending per capita*	0.57	0.53	0.49	0.32	0.41	−0.18 (0.31)	−0.19 (0.29)	−0.21 (0.30)	−0.35 (0.34)	−0.23 (0.28)
DAH per capita	0.41	0.86	0.85	0.38	0.84	0.28 (0.33)	0.06 (0.32)	0.06 (0.33)	0.34 (0.38)	0.06 (0.31)
DAH per birth on newborn and child health	0.77	0.97	0.87	0.47	0.93	0.18 (0.61)	0.02 (0.57)	0.09 (0.59)	0.50 (0.68)	0.05 (0.55)
DAH per birth on newborn and child health vaccines	0.15	0.076	0.20	0.33	0.12	0.86 (0.57)	0.98 (0.52)	0.74 (0.56)	0.67 (0.67)	0.83 (0.51)
Total spending per birth on routine immunisation vaccines	**0.0033**	**0.0007**	**0.014**	**0.029**	**0.0007**	1.12 (0.33)	1.18 (0.29)	0.94 (0.34)	1.00 (0.42)	1.14 (0.28)
Government spending per birth on routine immunisation vaccines	0.052	**0.020**	**0.034**	**0.024**	**0.016**	0.56 (0.27)	0.58 (0.22)	0.60 (0.26)	0.78 (0.31)	0.57 (0.21)

Each financial indicator was fitted in a logistic-logarithmic transformed fixed-effects regression model. P values below 0.05 are highlighted in bold.

*Private health spending is the sum of out-of-pocket and prepaid private health spending.

DAH, development assistance for health; DTP, diphtheria-tetanus-pertussis; GDP, gross domestic product; GNI, gross national income; MCV, measles-containing vaccine; Pol3, third dose of polio vaccine.

The country grouping criteria used by the mixed-effects models generated groups LIC+, LIC− and LMIC. During 2014–2018, LIC+ had a mean DTP3 coverage of 94%; surpassing LIC− (72%) and LMIC (85%). Table 2 shows the vaccination coverage summary for other mandatory vaccines proposed by WHO.^15^ Every country in LIC+ outperformed the mean coverage of LIC− and LMIC for each studied mandatory vaccine, showing that LIC+ have outstanding vaccination coverage overall and not limited to outstanding DTP3 coverage.

**Table 2:** Mean and SE of vaccination coverage, 2014–2018

Vaccine	Mean (SE) vaccination coverage %		
	LIC+	LIC−	LMIC
DTP1	96.9 (0.6)	81.8 (2.9)	90.2 (1.8)
DTP3	93.9 (1.1)	71.7 (3.9)	85.0 (2.5)
MCV1	91.8 (2.0)	69.4 (3.6)	85.2 (2.1)
BCG	96.1 (1.0)	80.6 (2.6)	90.1 (1.7)
Pol3	93.2 (1.1)	70.9 (3.7)	85.1 (2.2)

DTP, diphtheria-tetanus-pertussis; LIC, low-income countries; LMIC, lower-middle income countries; MCV, measles-containing vaccine; Pol3, third dose of polio vaccine.

For the mixed-effects models, we prioritised the analysis of financial indicators that were significant predictors of vaccination coverage, as indicated by the cross-sectional analysis (table 1). We fitted the year trends of government health spending per capita and total/government spending per birth on routine immunisation vaccines. We also included DAH per birth on newborn and child health vaccines in the analysis for being close to the p=0.05 threshold. The other financial indicators were also fitted into the mixed-effects models and their results can be found in the online supplemental materials.

**Figure 1:**
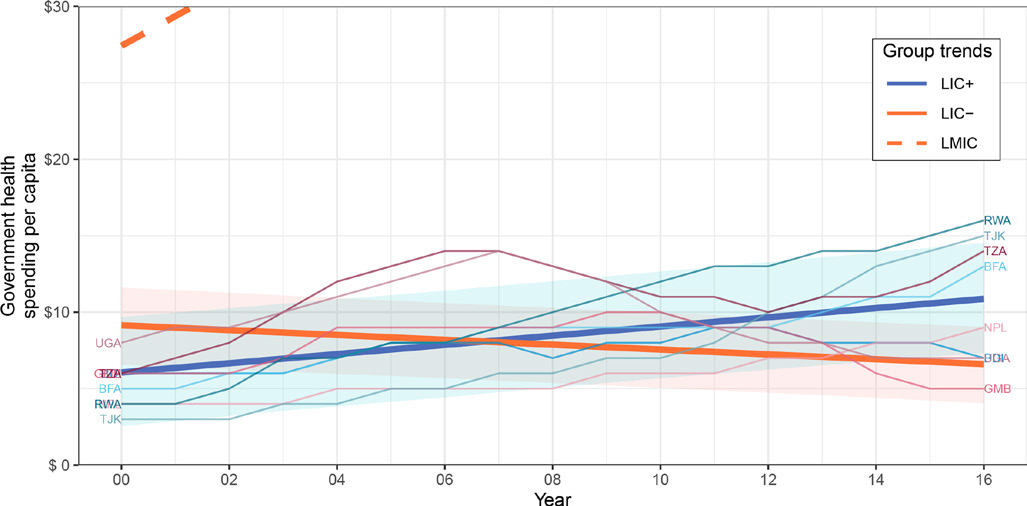
Government health spending per capita of country groups. Data source: IHME1. Indicator is in constant 2018 US$. The trends of LIC+, LIC− and LMIC were fitted by linear mixed-effects models; note most of LMIC trend was cut off for visibility. CIs of 95% surround LIC+ and LIC− trends and were computed through a parametric bootstrap method for mixed-effects models (10 000 simulations). LIC+ countries (ISO3): Burundi (BDI), Burkina Faso (BFA), Gambia (GMB), Nepal (NPL), Rwanda (RWA), Tajikistan (TJK), Tanzania (TZA) and Uganda (UGA). IHME, Institute for Health Metrics and Evaluation; LIC, low-income countries; LMIC, lower-middle income countries.

**Figure 2:**
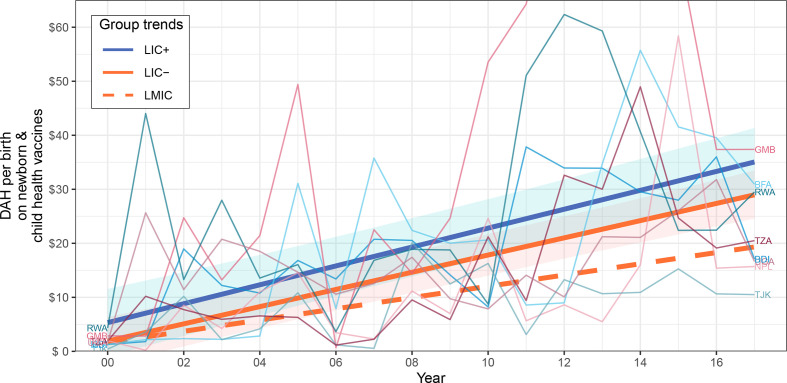
DAH per live birth on newborn and child health vaccines of country groups. Data source: IHME2. Indicator is in constant 2018 US$. The trends of LIC+, LIC− and LMIC were fitted by linear mixed-effects models. CIs of 95% surround LIC+ and LIC− trends and were computed through a parametric bootstrap method for mixed-effects models (10 000 simulations). LIC+ countries (ISO3): Burundi (BDI), Burkina Faso (BFA), Gambia (GMB), Nepal (NPL), Rwanda (RWA), Tajikistan (TJK), Tanzania (TZA) and Uganda (UGA). DAH, development assistance for health; IHME, Institute for Health Metrics and Evaluation; LIC, low-income countries; LMIC, lower-middle income countries.

**Figure 3:**
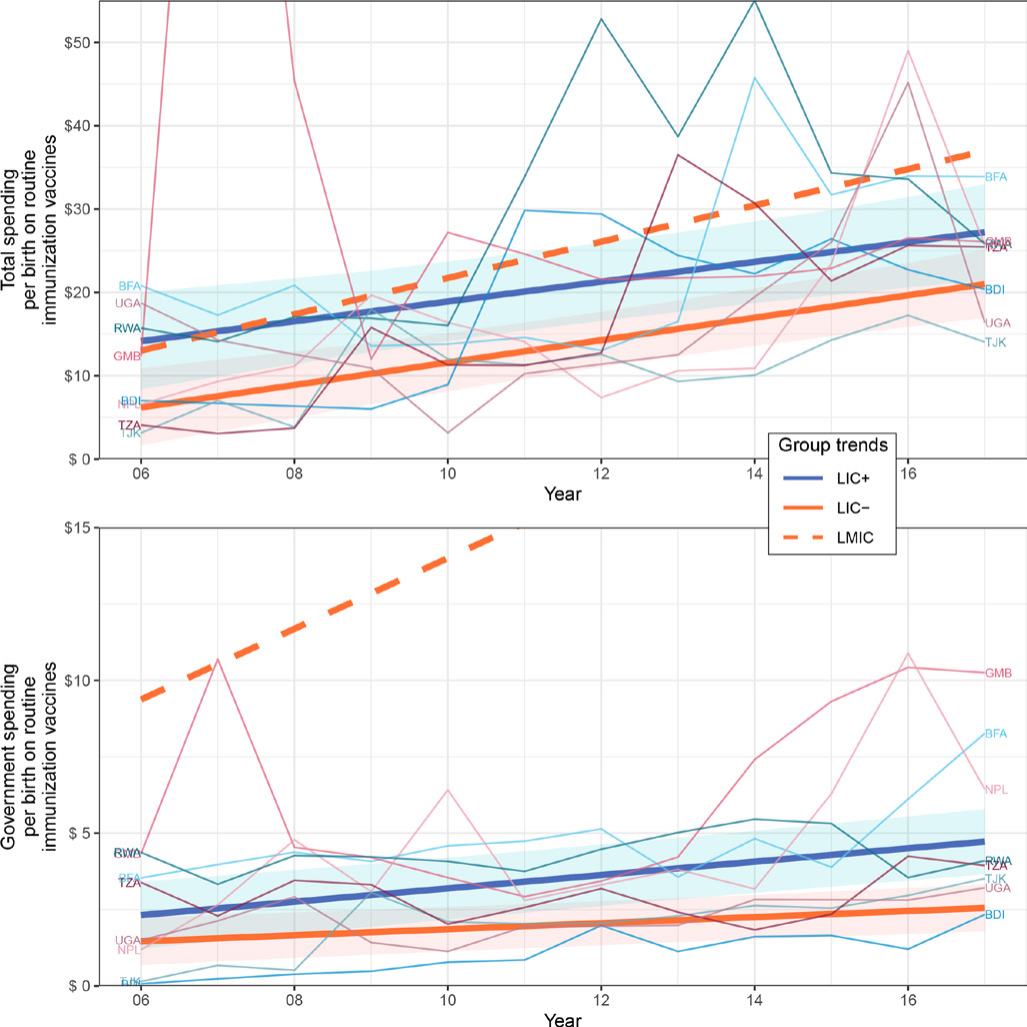
Total/government spending per birth on routine immunisation vaccines of country groups. Data source: WU2. Indicators are in constant 2010 US$. The trends of LIC+, LIC− and LMIC were fitted by linear mixed-effects models; note part of LMIC trend was cut off for visibility. CIs of 95% surround LIC+ and LIC− trends and were computed through a parametric bootstrap method for mixed-effects models (10 000 simulations). LIC+ countries (ISO3): Burundi (BDI), Burkina Faso (BFA), Gambia (GMB), Nepal (NPL), Rwanda (RWA), Tajikistan (TJK), Tanzania (TZA) and Uganda (UGA). LIC, low-income countries; LMIC, lower-middle income countries.

Table 3 summarises the intercept and slope (yearly rate of change) coefficients for each financial indicator and shows the p values of the country group comparisons to test if their indicator trends were significantly different. Figures 1–3 show the trends of the most relevant financial indicators studied; additional plots can be found in the online supplemental material for the other financial indicators.

**Table 3:** Summary of financial indicators’ rate of change of country groups

Indicator (US$)	Year range	Estimated intercept coefficients		Estimated slope coefficients and p values					Trend comparison p values		
		LIC+ α_00_	LIC− α_00_+α_01_	LIC+ α_10_	LIC− α_10_+α_11_	χ^2^	KR	PB	χ^2^	KR	PB
Government health spending per capita*	2000–2016	6.06 (1.81)	9.14 (2.22)	0.30 (0.05)	−0.16 (0.06)	**<0.0001**	**<0.0001**	**<0.0001**	**<0.0001**	**<0.0001**	**<0.0001**
DAH per birth on newborn and child health vaccines*	2000–2017	5.32 (3.20)	1.90 (3.92)	1.75 (0.19)	1.59 (0.24)	0.50	0.51	0.50	0.28	0.31	0.30
Total spending per birth on routine immunisation vaccines†	2006–2017	14.17 (2.97)	6.30 (3.79)	1.19 (0.32)	1.34 (0.42)	0.72	0.72	0.72	0.057	0.063	0.068
Government spending per birth on routine immunisation vaccines†		2.32 (0.55)	1.46 (0.68)	0.22 (0.04)	0.10 (0.05)	**0.0090**	**0.0093**	**0.0087**	**0.0021**	**0.0038**	**0.0033**

Each financial indicator was fitted by a linear mixed-effects regression model. The table shows the intercept and slope coefficients of LIC+ (α_00_ and α_10_) and LIC− (α_00_+α_01_ and α_10_+α_11_) with their SE in parentheses. χ^2^, KR and PB represent the p values of an asymptotic χ^2^ test, a Kenward-Roger approximation for F tests for reduction of mean structure and a parametric bootstrap method (10 000 simulations), respectively.^25^ There are two types of p values presented: the first corresponds to the significance of the slope coefficient α_11_, that is, if the yearly rate of change of LIC+ is significantly different from the yearly rate of change of LIC−. The second type of p values compare the overall trends of LIC+ and LIC−, that is, it does not refer to the significance of a specific coefficient but to the significance of intercept and slope combined. P values below 0.05 are highlighted in bold.

*Constant 2018 US$.

†Constant 2010 US$.

DAH, development assistance for health; LIC, low-income countries.

Government health spending per capita of LIC+ and LIC− had a significantly different rate of change (p<0.0001), increasing yearly for LIC+ by US$0.30 while decreasing yearly for LIC− by US$0.16. Figure 1 shows the government health spending per capita trends for country groups LIC+, LIC− and LMIC during 2000–2016. LMIC had the highest government health spending per capita, while all LIC+ countries eventually surpassed the LIC− trend except for Gambia.

The trends and rate of change of DAH per birth on newborn and child health vaccines were not statistically different between LIC+ and LIC− (p>0.28) although LIC+ appears to be slightly above LIC− as seen in figure 2. LIC+ and LIC− had similar rate of change of US$1.19 and US$1.34 per year, respectively.

The LIC+ trend began with a higher total spending per birth on routine immunisation vaccines than LIC−, of US$14.17 and US$6.30, respectively, although LIC+ increased at a slower rate of US$1.19 per year as opposed to US$1.34 per year increase in LIC−. The difference between LIC+ and LIC− overall trends was almost statistically significant (p>0.057), something expected considering the high variability in spending between countries and over time. The trends and rate of change of government spending per birth on routine immunisation vaccines were significantly different between LIC+ and LIC− (p<0.0093) where LIC+ always lead above LIC− with a yearly rate of change of US$0.22 as opposed to US$0.10. Figure 3 shows the per live birth total spending and government spending on routine immunisation vaccines for country groups LIC+, LIC− and LMIC during 2006–2017. LMIC had the highest total spending per live birth on routine immunisation vaccines.

## Discussion

Our analyses revealed that performance differences in vaccination coverage among LIC could not be explained by the countries’ economic development (GDP/GNI per capita), total health spending per capita, nor aggregated DAH per capita. Other studies have also observed that higher health spending does not always results in improved health services or outcomes.^26^ On the other hand, government health spending per capita and total/government spending per birth on routine immunisation vaccines were significant positive predictors of vaccination coverage in LIC. Other studies have found similar relationships in which higher government spending combined with low out-of-pocket spending were associated with higher national vaccination coverage.^27^

An increasing rate of government health spending per capita could explain the vaccination performance of LIC+. The government health spending per capita of LIC+ increased over time while it decreased for LIC−; all except one country in LIC+ reached or exceeded the government health spending trend of LIC− by 2016. This finding underscores the importance of government commitment in LIC to improve vaccination coverage, child health and healthcare in general, making vaccines and healthcare accessible to more people. For example, previous research found that the success of Rwanda’s vaccine programme was multifactorial, where one of the main factors was a strong and high-level political will.^28^ Nepal was the first LIC to have a national newborn strategy, influencing similar strategies in other countries; this was made possible due to political commitment that supported newborn survival.^29^

Both the total and government spending per birth on routine immunisation vaccines were significant predictors of vaccination coverage. This strengthens the idea that government plus DAH investments directly into routine immunisation can improve vaccination coverage. DAH on vaccines also partly explain the difference in vaccination performance between LIC+ and LIC−; although the evidence is less conclusive than for the previously mentioned indicators. LIC+ received slightly more DAH per live birth on newborn and child health vaccines than LIC−, but these trends were not statistically different, possibly in part due to the following two factors: (1) the large variation in DAH on vaccines year-to-year or across the LIC+countries, and (2) DAH funding that was used for purposes different to routine immunisation such as introduction of new vaccines, health system strengthening and supplementary activities. For example, the introductions of the pneumococcal vaccine in Malawi in November 2011^30^ and Nepal in January 2015^31^ generated huge spikes of DAH on newborn and child health vaccines that were not aimed towards routine immunisation. As previous studies have suggested, LIC will remain dependent on DAH in the near future, unless they increase government health spending substantially.^11^

## Conclusion

Our analysis suggests that an increasing government health spending per capita—with an increasing government spending per birth on routine immunisation vaccines—and DAH on vaccines may have led to an efficient utilisation of healthcare resources and the immunisation success of LIC+. The financial commitment of LIC+ governments was clear as their health spending increased over time, as opposed to LIC− that decreased/stagnated. Government health funds for routine vaccination also increased more rapidly in LIC+ than LIC−. Funders that actively invest into countries or programmes to improve vaccination coverage might want to consider the countries’ government health spending levels when making investment decisions. LIC continue to be dependent on DAH to achieve high vaccination coverage and remain far behind in government health spending on vaccines when compared with LMIC.

## Data Availability

Data are available from public, open access repositories. WHO-UNICEF. WHO and UNICEF estimates of national infant immunisation coverage, 2019. https://data.unicef.org/resources/dataset/immunization. World Bank. World development indicators, 2019. https://data.worldbank.org. IHME. Global health spending 1995–2016, 2019. http://ghdx.healthdata.org/record/ihme-data/global-health-spending-1995-2016. IHME. Development assistance for health database 1990–2018, 2019. http://ghdx.healthdata.org/record/ihme-data/development-assistance-health-database-1990-2018. WHO-UNICEF. WHO-UNICEF joint reporting form: immunisation financing indicators, 2019. https://www.who.int/immunization/programmes_systems/financing/data_indicators/en
