# Supplementary material for: Health spending and vaccination coverage in low-income countries
Source: BMJ Glob Health. 2021 May 5;6(5):e004823. doi: 10.1136/bmjgh-2020-004823 (PMC8103936; doi:10.1136/bmjgh-2020-004823)
Supplement: Supplementary data [file bmjgh-2020-004823supp001.pdf]

Supplemental Materials: Health spending and vaccination coverage in low-income countries

Francisco Castillo-Zunino,[a] Pinar Keskinocak,[a] Dima Nazzal,[a] Matthew C Freeman[b]
[a] H. Milton Stewart School of Industrial and Systems Engineering, Georgia Institute of Technology, Atlanta, GA, USA.
[b] Rollins School of Public Health, Emory University, Atlanta, GA, USA.

Tables

Table 1: Summary of data sources

Data source		Variable	Missing data %	Time period
Short	Full			
WU1[1]	WHO-UNICEF: estimates of national infant immunization coverage	DTP1 coverage<br>DTP3 coverage<br>MCV1 coverage<br>BCG coverage<br>Pol3 coverage	0<br>0<br>0<br>0<br>0	2000–18
WB1[2]	World Bank: world development indicators	GNI per capita<br>GDP per capita<br>Population<br>Land area	0<br>0<br>0<br>0	2000–18
		Live birth rate	0	2000–17
UNDP1[3]	UN Development Programme: human development reports	Mean years of schooling, female	4.2	2014–18
IHME1[4]	IHME: global health spending 1995–2016	Total health spending per capita<br>Government health spending per capita<br>Out-of-pocket health spending per capita<br>Prepaid private health spending per capita<br>DAH per capita	0<br>0<br>0<br>0<br>0	2000–16
IHME2[5]*	IHME: development assistance for health database 1990–2018	DAH per live birth on newborn & child health<br>DAH per live birth on newborn & child health vaccines	0<br>0	2000–17
WU2[6]*	WHO-UNICEF joint reporting form: immunization financing indicators	Total spending per live birth on routine immunization vaccines<br>Government spending per live birth on routine immunization vaccines	10.1<br>5.2	2006–17

** With data source WB1 we calculated per live birth values (using population and live birth rate). Missing data is calculated as the percentage of country-year samples missing among LIC data for the specified time period.*

Castillo-Zunino F, *et al. BMJ Global Health* 2021; 6:e004823. doi: 10.1136/bmjgh-2020-004823

Table 2: Country groups with ISO3 codes by Gavi co-financing transition status (2018):

**LIC+**				**LIC-**					
**Initial self-financing:**		**Preparatory transition:**		**Initial self-financing:**				**Preparatory transition:**	
Burundi	BDI	Tajikistan	TJK	Benin	BEN	Mali	MLI	Yemen, Rep.	YEM
Burkina Faso	BFA			Central African Rep.	CAF	Mozambique	MOZ		
Gambia	GMB			Congo, Dem. Rep.	COD	Malawi	MWI		
Nepal	NPL			Ethiopia	ETH	Niger	NER		
Rwanda	RWA			Guinea	GIN	Sierra Leone	SLE		
Tanzania	TZA			Guinea-Bissau	GNB	Chad	TCD		
Uganda	UGA			Haiti	HTI	Togo	TGO		
				Madagascar	MDG				

**LMIC**									
**Initial self-financing:**		**Preparatory transition:**		**Accelerated transition:**		**Fully self-financing:**		**Not Gavi eligible:**	
Senegal	SEN	Bangladesh	BGD	Angola	AGO	India	IND	Egypt, AR	EGY
Zimbabwe	ZWE	Côte d'Ivoire	CIV	Bolivia	BOL	Lao PDR	LAO	Morocco	MAR
		Cameroon	CMR	Congo, Rep.	COG	Nigeria	NGA	Philippines	PHL
		Ghana	GHA	Honduras	HND	Nicaragua	NIC	Palestinian Territory	PSE
		Kenya	KEN	Indonesia	IDN	Papua New Guinea	PNG	El Salvador	SLV
		Kyrgyz Rep.	KGZ	Moldova, Rep.	MDA	Uzbekistan	UZB	Swaziland	SWZ
		Cambodia	KHM	Mongolia	MNG	Vietnam	VNM	Tunisia	TUN
		Lesotho	LSO					Ukraine	UKR
		Mauritania	MRT						
		Pakistan	PAK						
		Sudan	SDN						
		Zambia	ZMB						

Castillo-Zunino F, *et al. BMJ Global Health* 2021; 6:e004823. doi: 10.1136/bmjgh-2020-004823

Table 3: Summary of other financial indicators' change rates of country groups

Indicator (US$)	Year range	Intercept coefficients		Slope coefficients & p-values					Trend comparison p-values		
		LIC+ α_{00}	LIC- $\alpha_{00}+\alpha_{01}$	LIC+ α_{10}	LIC- $\alpha_{10}+\alpha_{11}$	χ^2	KR	PB	χ^2	KR	PB
GDP per capita[a]	00-18	224 (65.4)	296 (80.1)	32.24 (1.68)	25.11 (2.06)	**0.0006**	**0.0006**	**0.0009**	**0.0026**	**0.0047**	**0.0046**
GNI per capita[b]	00-18	214 (61.43)	264 (75.23)	33.27 (1.67)	25.87 (2.04)	**0.0003**	**0.0003**	**0.0007**	**0.0015**	**0.0029**	**0.0032**
Total health spending per capita[c]	00-16	22.7 (5.89)	28.6 (7.22)	1.37 (0.14)	0.73 (0.17)	**0.0001**	**0.0002**	**0.0002**	**0.0007**	**0.0016**	**0.0018**
Private spending per capita[c]	00-16	12.36 (4.44)	16.61 (5.44)	0.32 (0.07)	0.19 (0.09)	0.14	0.15	0.15	0.28	0.30	0.30
DAH per capita[c]	00-16	4.39 (2.08)	2.93 (2.54)	0.74 (0.09)	0.69 (0.11)	0.65	0.65	0.65	0.65	0.67	0.67
DAH per birth on newborn & child health[c]	00-17	5.21 (7.64)	1.31 (9.36)	4.35 (0.44)	4.59 (0.54)	0.65	0.66	0.65	0.88	0.88	0.88

Each financial indicator was fitted by a linear mixed-effects regression model. The table shows the intercept and slope coefficients of LIC+ (α_{00} and α_{10}) and LIC- ($\alpha_{00}+\alpha_{01}$ and $\alpha_{10}+\alpha_{11}$) with their standard error in parentheses. χ^2, KR, and PB represent the p-values of an asymptotic χ^2 test, a Kenward-Roger approximation for F tests for reduction of mean structure, and a parametric bootstrap method (10,000 simulations) respectively[7]. There are two types of p-values presented: the first corresponds to the significance of the slope coefficient α_{11}, i.e., if the yearly change rate of LIC+ is significantly different from the yearly change rate of LIC-. The second type of p-values compare the overall trends of LIC+ and LIC-, i.e., it does not refer to the significance of a specific coefficient but to the significance of intercept and slope combined. p-values below 0.05 are highlighted in bold.

[a] *current US$.*

[b] *US$ using World Bank Atlas method.*

[c] *constant 2018 US$.*

Code implementation

We implemented all linear mixed-effects models with the R library *lme4*[8] by running the code

$$lmer(var \sim year * group + (1|country), data = sample) \quad (1)$$

where *var* represents the variable being fitted over time, *sample* represents the set with all data points, and the rest represent the *year*, *country*, and *group* (LIC+, LIC-, or LMIC) of the data points.

To compare the slope coefficients (change rate per year) between LIC+ and LIC-, we used the following code in R to calculate p-values using library *pbkrtest*[7]:

$$model1 = lmer(var \sim year * group + (1|country), data = sample\ ) \quad (2)$$

$$model2 = lmer(var \sim year + group + (1|country), data = sample\ ) \quad (3)$$

$$anova(model1, model2) \quad (4)$$

$$KRmodcomp(model1, model2) \quad (5)$$

$$PBmodcomp(model1, model2, nsim = 10000) \quad (6)$$

In this case, *sample* only includes LIC. Line (2) defines the complete mixed-effects model that considers intercept and slopes for each country group (LIC+ and LIC-) with mixed-effects for each country. Line (3) shows a reduced version of the first model where country groups have different intercepts but share the same slope – comparing both models tells you if group specific slopes are statistically relevant or not (corresponds to the p-value of coefficient α_{11} as defined in the paper). Both models in (2) and (3) are compared in lines (4), (5), and (6) by computing the asymptotic χ^2 test, the Kenward-Roger approximation, and parametric bootstrap[7], respectively. Line (6) runs 10,000 parametric bootstrap replications to obtain a good estimate.

We also compared the overall trends between LIC+ and LIC-, namely compared both the intercept and slope coefficients of country groups simultaneously. The code is almost identical to (2)-(6), except for line (3) that was replaced by:

$$model2 = lmer(var \sim year + (1|country), data = sample\ ) \quad (7)$$

Line (7) shows a reduced version of the model in line (2), where there is no grouping coefficients only shared intercept and slope.

To calculate and plot the *95%* confidence intervals of LIC+ and LIC- trends we used function *bootMer (from lme4* library[8]) with 10,000 parametric bootstrap simulations.

Additional figures

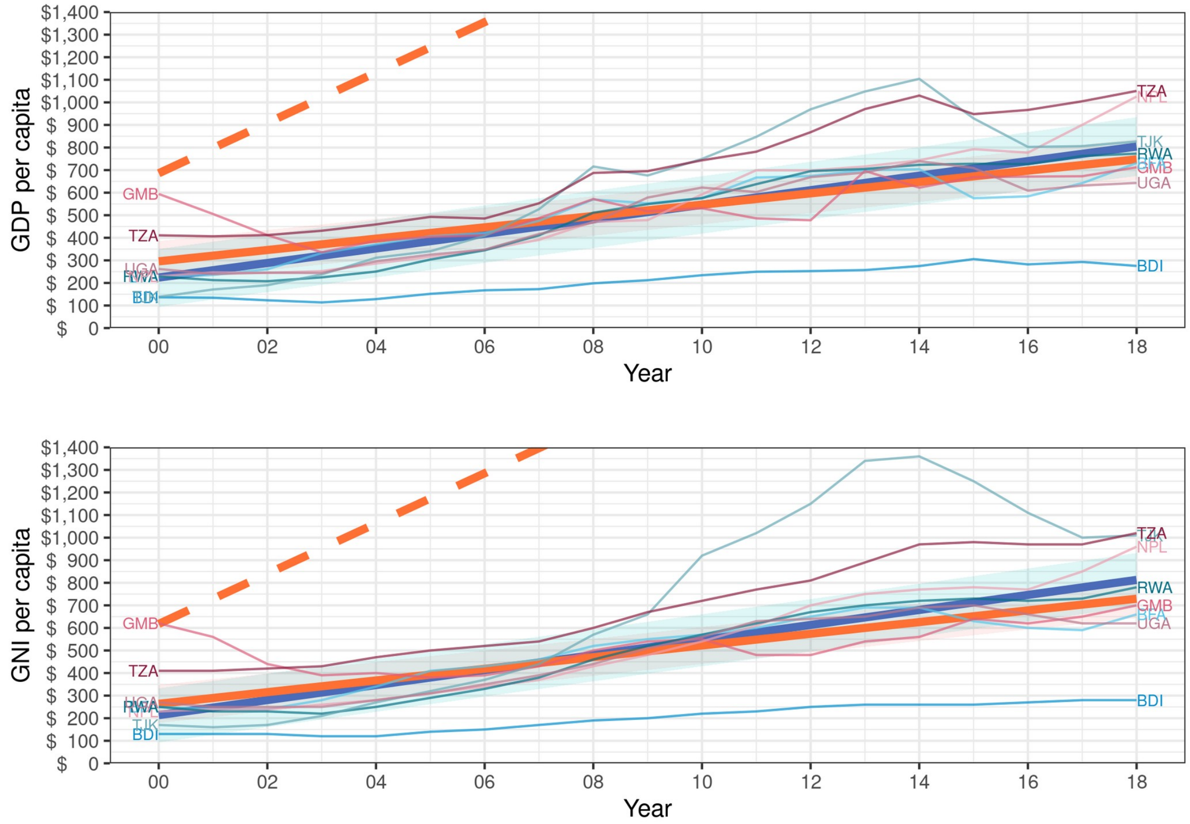

Figure 1: GDP & GNI per capita of country groups

Data source: WB1. GDP per capita is in current US$, GNI per capita is in US$ using World Bank Atlas method. The trends of LIC+, LIC-, and LMIC were fitted by linear mixed-effects models; note part of LMIC trends were cut off for visibility. Confidence intervals of 95% surround LIC+ and LIC- trends and were computed through a parametric bootstrap method for mixed-effects models (10,000 simulations). LIC+ countries (ISO3): Burundi (BDI), Burkina Faso (BFA), Gambia (GMB), Nepal (NPL), Rwanda (RWA), Tajikistan (TJK), Tanzania (TZA), and Uganda (UGA).

Castillo-Zunino F, *et al. BMJ Global Health* 2021; 6:e004823. doi: 10.1136/bmjgh-2020-004823

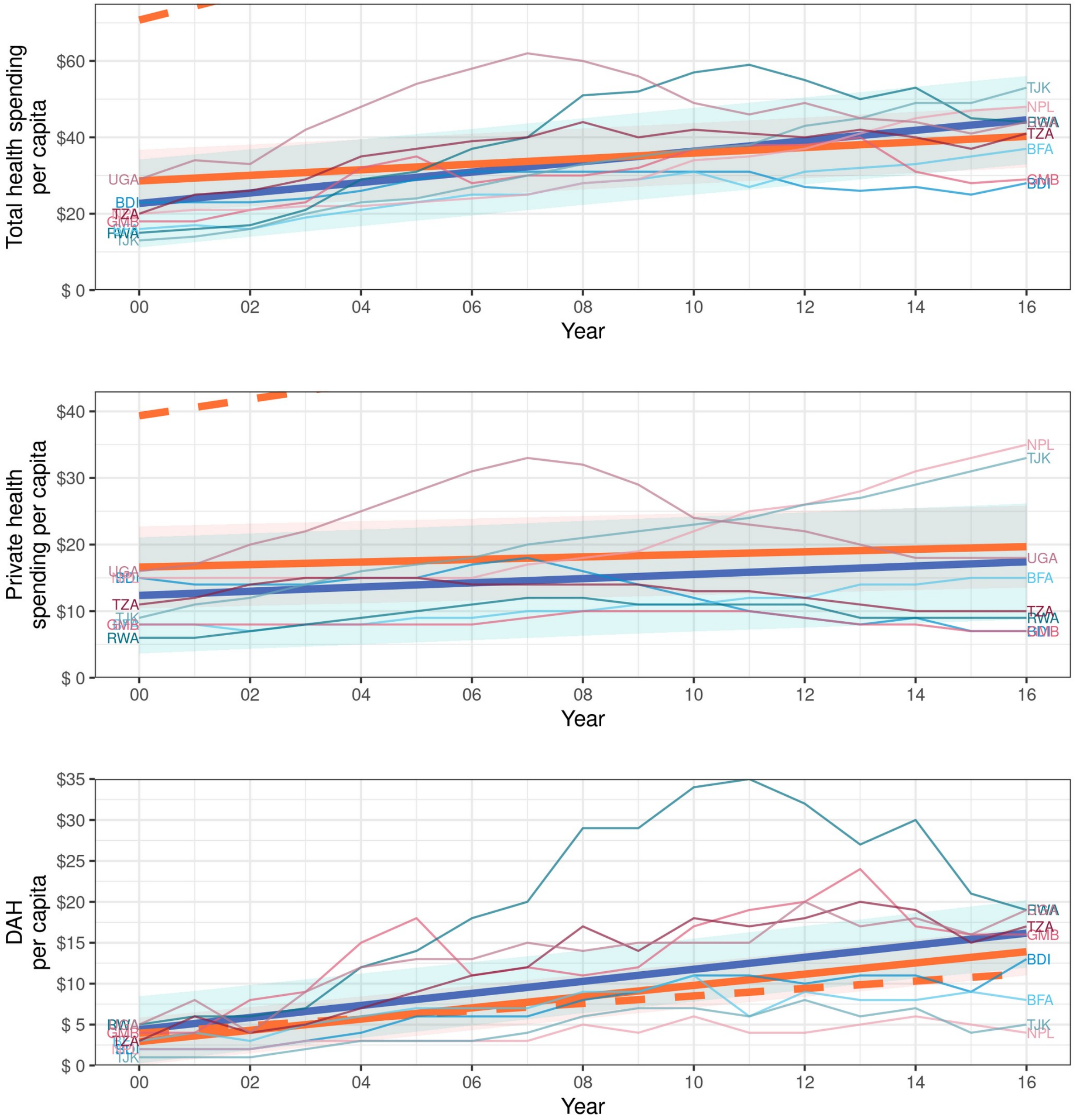

Figure 2: Total/private health spending and DAH per capita of country groups

Data source: IHME1. All spendings are in constant 2018 US$. The trends of LIC+, LIC-, and LMIC were fitted by linear mixed-effects models; note part of LMIC trends were cut off for visibility. Confidence intervals of 95% surround LIC+ and LIC- trends and were computed through a parametric bootstrap method for mixed-effects models (10,000 simulations). LIC+ countries (ISO3): Burundi (BDI), Burkina Faso (BFA), Gambia (GMB), Nepal (NPL), Rwanda (RWA), Tajikistan (TJK), Tanzania (TZA), and Uganda (UGA).

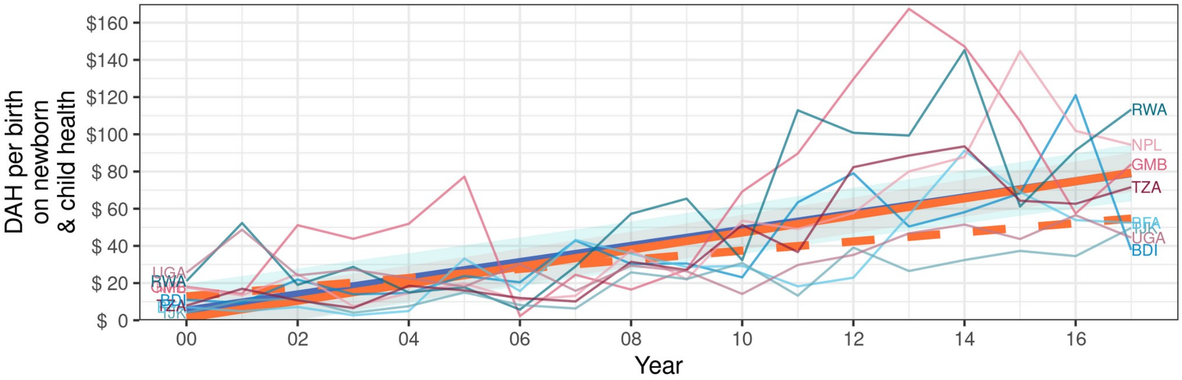

Figure 3: DAH per birth on newborn & child health of country groups

Data source: IHME2. Indicator is in constant 2018 US$. The trends of LIC+, LIC-, and LMIC were fitted by linear mixed-effects models. Confidence intervals of 95% surround LIC+ and LIC- trends and were computed through a parametric bootstrap method for mixed-effects models (10,000 simulations). LIC+ countries (ISO3): Burundi (BDI), Burkina Faso (BFA), Gambia (GMB), Nepal (NPL), Rwanda (RWA), Tajikistan (TJK), Tanzania (TZA), and Uganda (UGA).

References

1 WHO-UNICEF. WHO and UNICEF estimates of national infant immunization coverage, 2019. https://data.unicef.org/resources/dataset/immunization/ (accessed Jan 27, 2020).
2 World Bank. World development indicators, 2019. https://data.worldbank.org (accessed Jan 27, 2020).
3 United Nations Development Programme. Human development reports, 2020. http://hdr.undp.org/en/data (accessed Mar 3, 2021).
4 IHME. Global health spending 1995–2016, 2019. http://ghdx.healthdata.org/record/ihme-data/global-health-spending-1995-2016 (accessed Jan 27, 2020).
5 IHME. Development assistance for health database 1990–2018, 2019. http://ghdx.healthdata.org/record/ihme-data/development-assistance-health-database-1990-2018 (accessed Jan 27, 2020).
6 WHO-UNICEF. WHO-UNICEF joint reporting form: immunization financing indicators, 2019. https://www.who.int/immunization/programmes_systems/financing/data_indicators/en/ (accessed Jan 27, 2020).
7 Halekoh U, Højsgaard S. A Kenward-Roger approximation and parametric bootstrap methods for tests in linear mixed models–the R package pbkrtest. *Journal of Statistical Software* 2014; **59**: 1–30.
8 Bates D, Machler M, Bolker B, Walker S. Fitting linear mixed-effects models using lme4. *arXiv preprint* 2014; arXiv:1406.5823.
9 Halekoh U, Højsgaard S. A Kenward-Roger approximation and parametric bootstrap methods for tests in linear mixed models–the R package pbkrtest. *Journal of Statistical Software* 2014; **59**: 1–30.
